# Early sclerostin expression explains bone formation inhibition before arthritis onset in the rat adjuvant-induced arthritis model

**DOI:** 10.1038/s41598-018-21886-w

**Published:** 2018-02-22

**Authors:** Guillaume Courbon, Raphaëlle Lamarque, Maude Gerbaix, Robin Caire, Marie-Thérèse Linossier, Norbert Laroche, Mireille Thomas, Thierry Thomas, Laurence Vico, Hubert Marotte

**Affiliations:** 1 SAINBIOSE, INSERM U1059, University of Lyon, Saint-Etienne, France; 2 Department of Rheumatology, Hopital Nord, University Hospital, Saint-Etienne, France

## Abstract

Periarticular bone loss in rheumatoid arthritis (RA) is considered to be mainly related to synovial inflammation. However, strong bone loss has also described at the time of arthritis onset. Recently, a paradoxical exacerbation of joint damage was described when blocking sclerostin in various arthritis models. Thus, we aimed to determine kinetics of bone loss and its mechanisms in the adjuvant induced arthritis (AIA) rat model of RA. AIA was induced (n = 35) or not (n = 35) at day 0. In addition to well-known arthritis at day 12, we showed with 3D-imaging and histomorphometry that bone microstructural alterations occurred early from day 8 post-induction, characterized by cortical porosity and trabecular bone loss. Active osteoclastic surfaces were increased from day 8 with RANKL upregulation. More surprisingly SOST and DKK1 were overexpressed from day 6 and followed by a dramatic decrease in bone formation from day 8. At the time of arthritis onset, SOST and DKK1 returned to control values, but frizzled related protein 1 (SFRP1), proinflammatory cytokines, and MMPs started to increase. Bone alterations before arthritis onset reinforce the hypothesis of an early bone involvement in arthritis. Kinetics of osteocyte markers expression should be considered to refine Wnt inhibitor treatment strategies.

## Introduction

Rheumatoid arthritis (RA) physiopathology includes synovial inflammation with proinflammatory cytokine overexpression of such as tumor necrosis factor (TNF), interleukin (IL)-1, IL-6, and IL-17^1^. These events induce joint swelling, the first clinical sign observed in arthritis and considered as the disease onset^2^. Subchondral bone loss is a linked phenomenon, mainly related so far to an increased bone resorption dependent on the receptor activator of nuclear factor kappa B ligand (RANKL) pathway^3,4^. Enhanced osteoclast number and activity have been identified to cause a local degradation defined as bone erosion, which widely contributes to loss of articular function and pain^5,6^. Osteoclast-mediated bone resorption includes enzymes such as cathepsin K (encoded by the CTSK gene) and tartrate-resistant acid phosphatase (TRAcP5 enzyme, encoded by the ACP5 gene). The common paradigm sets subchondral bone change as a consequence of joint inflammation and pannus formation^7,8^. Some new insights have reported bone erosion in the periarticular area of metacarpophalangeal joints in RA patients during the first two years following diagnosis^9^. Recently, erosion was also described in patients positive with anticitrullinated protein antibodies (ACPA), a RA early marker without clinical joint inflammation^10^, but with tenosynovitis, synovitis, and osteitis on magnetic resonance imaging^11^. However despite these abnormalities described in the preclinical RA phase, the RA onset is defined by joint effusion occurring^2^.

In the female Lewis rat adjuvant-induced arthritis (AIA) model, ankle bone porosity was increased with trabecular bone loss at the day of clinical arthritis onset, occurring mainly at the navicular bone site^12^. Furthermore, these early signs predicted later inflammation and bone loss outcome. All these elements are in favor of early involvement of the bone compartment, as suggested by the Inside-Outside hypothesis^7,8^. Another major insight of the last years is the involvement of bone formation inhibition mediated by the Wnt pathway during arthritis^13^. Dickkopf 1 (DKK1), sclerostin (encoded by the SOST gene), and secreted frizzled related protein 1 (SFRP1) are three upstream extracellular Wnt pathway inhibitors. DKK1 and sclerostin block low density lipoprotein receptor related (LRP) 5/6 coreceptors of Frizzled in canonical Wnt signalling transduction, while SFRP1 can bind directly Wnt and Frizzled^14^.

DKK1 was increased in RA serum and correlated with bone structure alterations, bone mineral density loss, and joint damage^15–18^. DKK1 knockout mice or antibody targeting DKK1 in animal models improved bone tissue during arthritis^19^. However, even if sclerostin inhibitors seems promising to treat osteoporosis^20^, its effect on bone during arthritis is more controversial. Firstly, sclerostin blockade in an animal model was described as beneficial for the bone during arthritis^21,22^. These studies were conducted in two arthritic animal models. The transgenic mice for human TNF (hTNFtg) were treated at a late stage of disease from weeks 8 to 11^21^, while prophylactic and therapeutic approaches were performed in collagen-induced arthritis mice^22^. On the contrary, novel insights in TNF-dependent mouse models suggested that sclerostin blockade might be deleterious for arthritis since SOST deficiency worsened arthritis in the combined mouse model of SOST^−/−^/hTNFtg^23^. In the same work, sclerostin blockade with neutralizing antibody into glucose-6-phosphate isomerase (G6PI) arthritis did not improve arthritis either. However, a loss of sclerostin ameliorated disease severity in the K/BxN serum transfer model. In humans, sclerostin serum level was decreased in early RA patients compared to controls^18^. SFRP1 was reported elevated in supernatants of RA synoviocytes^24^. However, its implication in RA pathogenesis remains under investigation and could promote Th17 differentiation of T cells^25^.

To investigate the clear role of Wnt inhibitors in relation to bone alteration in the early phases of arthritis, we explored periarticular cortical and trabecular bone microarchitecture at 7 different time points, particularly before arthritis onset, and correlated the bone alteration with gene expressions focusing on Wnt inhibitors in the rat AIA model.

## Results

### Arthritis was observed from day 12

As expected, arthritis was detected at day 12 (Figure S1) with a strong increase of articular index (AI), ankle circumferences, and loss of function index (LFI) in the AIA group compared to control (CTRL) group (Figure S1A–C, respectively, p < 0.001 each). Body mass was decreased in the AIA group from day 12 compared to CTRL group (Figure S1D, p < 0.01). At later stage between day 17 and day 24, joint inflammation parameters assessed by AI and ankle circumferences decreased in the AIA group (p < 0.05 and p < 0.01, respectively), while LFI remained high. No inflammatory cells infiltrate was observed at the ankle site in histology at day 8 (Figure S1E). Accordingly to these results, we named arthritis phase the period after clinical arthritis onset (from day 12 to day 24) and pre-arthritis phase corresponded to time points without clinical sign (day 0 of induction until day 10).

### Cortical bone deterioration started before AIA onset

**Figure 1:**
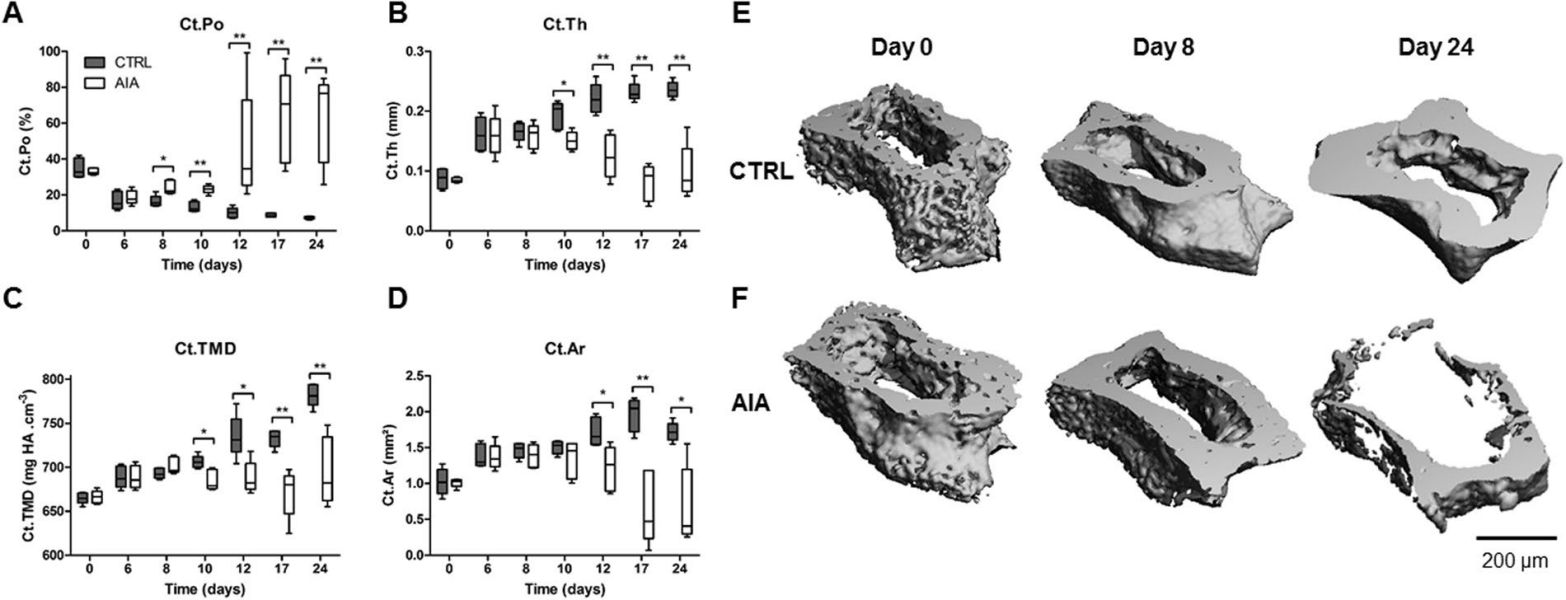
Cortical alterations of navicular bone in the rat AIA model compared to control. Microcomputed tomography analysis of the cortical layer with (**A**) Ct.Po, (**B**) Ct.Th, (**C**) Ct.TMD, and (**D**) Ct.Ar represented at various time points before and after arthritis onset. 3-D imaging illustrating Ct.Po changes in CTRL (**E**) versus AIA (**F**) groups at day 0, 8 and 24. Scale bar: 200 µm. AIA: adjuvant-induced arthritis (n = 5 at each time point); CTRL: control (n = 5 at each time point); Ct.Po: cortical porosity; Ct.Th: cortical thickness; Ct.TMD: cortical tissue mineral density (mg of hydroxyapatite per cm^3^), Ct.Ar: cortical area. Boxes and bars were median ± interquartile range. Mann-Whitney test: *p < 0.05 and **p < 0.01.

Cortical porosity (Ct.Po) was the earliest structural cortical parameter to be altered from day 8 (Fig. 1A, p < 0.05). Then, cortical thickness (Ct.Th) and Ct. tissue mineral density (TMD) decreased in the AIA group compared to CTRL group from day 10 (Fig. 1B,C, p < 0.05). After day 12, Ct. area (Ar) was lower in the AIA group compared to CTRL group (Fig. 1D, p < 0.05). Three-dimensional illustrations supported these results (Fig. 1E,F**)**.

### Trabecular bone loss occurred before AIA onset

**Figure 2:**
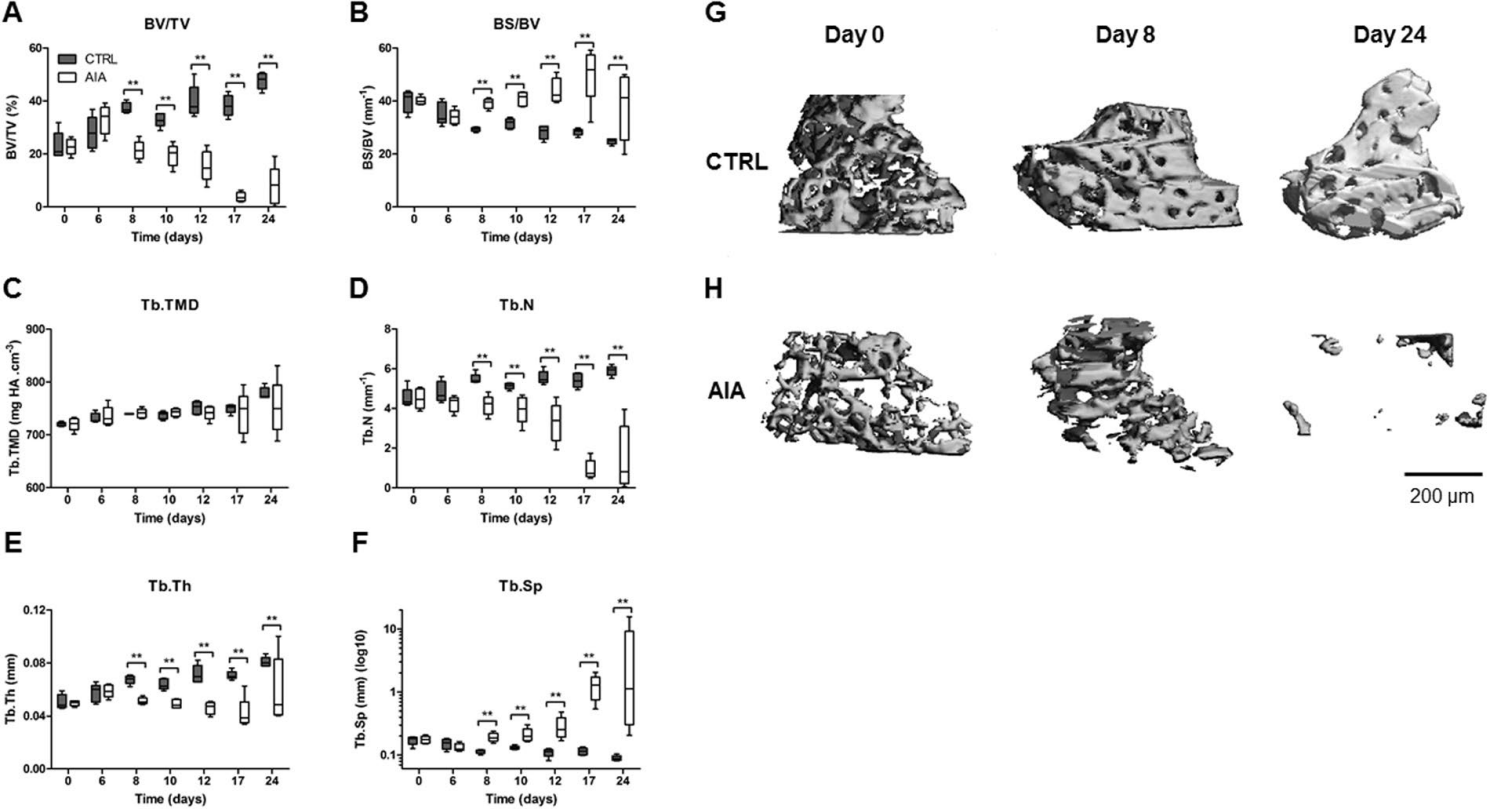
Trabecular changes of navicular bone in the rat AIA model compared to control. Microcomputed tomography study of the trabecular network with (**A**) BV/TV, (**B**) BS/BV, (**C**) Tb.TMD, (**D**) Tb.N, (**E**) Tb.Th, and (**F**) Tb.Sp represented at the same time points. 3-D imaging illustrating trabecular changes in CTRL (**G**) versus AIA (**H**) rats groups at day 0, 8 and 24. Scale bar: 200 µm. AIA: adjuvant-induced arthritis (n = 5 at each time point); CTRL: control (n = 5 at each time point); BV/TV: bone volume /tissue volume; BS/BV: bone surface/bone volume; Tb.TMD: trabecular tissue mineral density; Tb.N: trabecular number; Tb.Th: trabecular thickness; Tb.Sp: trabecular separation. Boxes and bars were median ± interquartile range. Mann-Whitney test: **p < 0.01.

Trabecular bone loss also occurred as early as day 8 with a decreased bone volume fraction, while bone surface fraction was increased in rat AIA compared to CTRL (Fig. 2A,B, p < 0.01). No change in trabecular (Tb.) TMD was observed over time (Fig. 2C). The Tb. number (N) and Tb. thickness (Th) decreased from day 8 (Fig. 2D,E, p < 0.01 each) with an increased Tb. separation (Sp) (Fig. 2F, p < 0.01) in the AIA group compared to CTRL group. Three-dimensional illustrations showed trabecular loss from day 8 (Fig. 2G,H**)**. Furthermore, the trabecular network was disorganized in the AIA group compared to CTRL group with increased structure model index (SMI) and anisotropy, and decreased connecting density (Conn.D.) (Figure S2A–C respectively, p < 0.05).

### Mechanisms associated to early bone loss were related to increased osteoclast activity and decreased osteoblast activity

**Figure 3:**
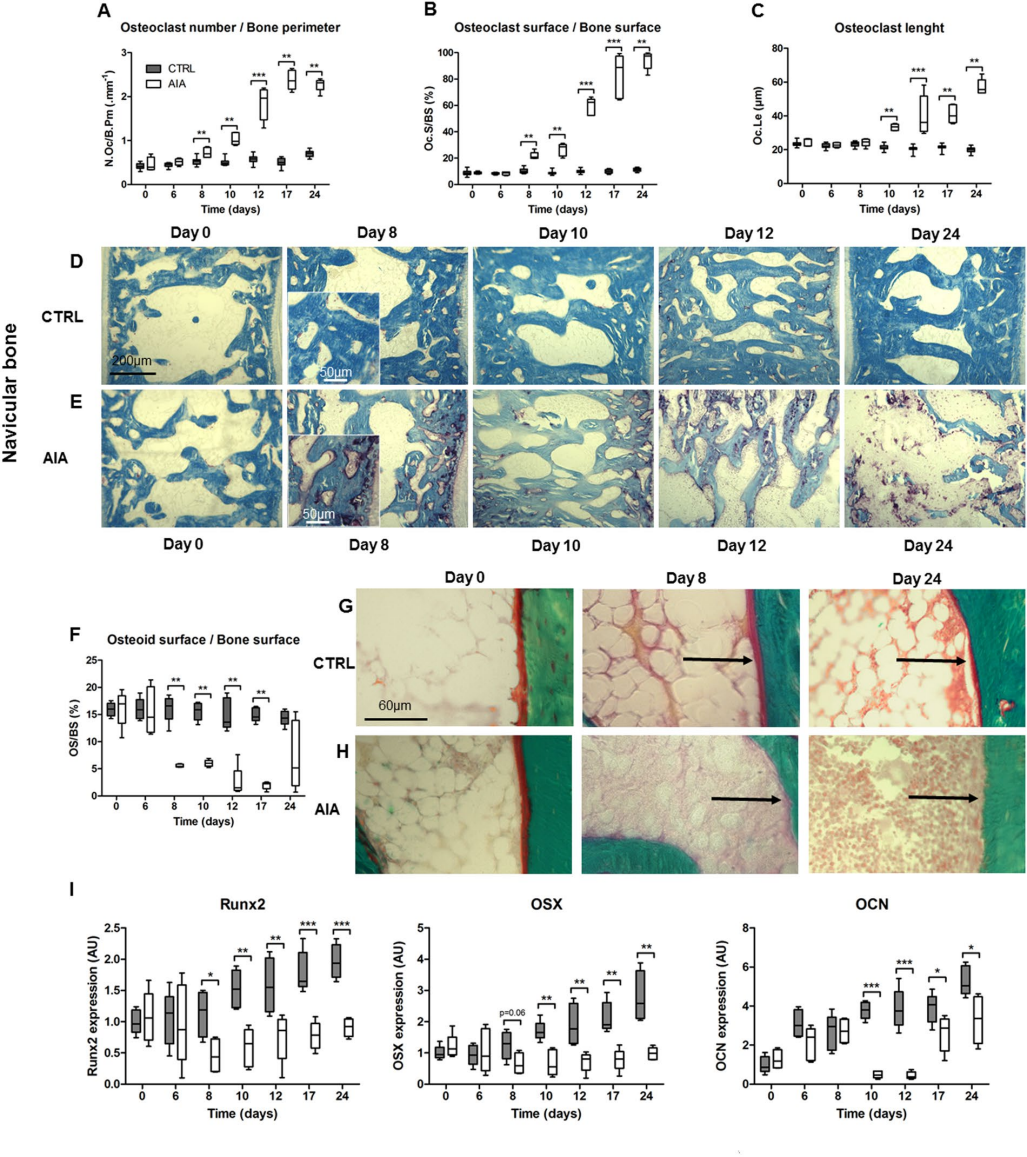
Early bone uncoupling was assessed: increased bone resorption and decreased mineralization were observed. Histological assessment of osteoclast with TRAcP5+ cell staining: (**A**) N.Oc/B.Pm, (**B**) Oc.S/BS, and (**C**) Oc.Le. Representative slices were provided from rats at days 0, 8, 10, 12 and 24 in CTRL (**D**) and AIA (**E**). Bone tissue and TRAcP5+ cells were stained in blue and purple, respectively. Scale bar: 200 µm, except for the inset (scale bar: 25 µm). (**F**) Osteoid matrix deposit on modified Goldner trichrome quantified with OS/BS, with representative slices from CTRL group (**G**) and AIA group (**H**). Osteoid layer along mineralized trabeculae and marrow cavity were stained in red, green, and yellow, respectively. Scale bar: 60 µm. (I) Osteoblastic markers decreased in the AIA group included RUNX2, OSX, and OCN. N.Oc/B.Pm: number of osteoclasts by bone perimeter; Oc.S/BS: osteoclast surface by bone surface; Oc.Le: osteoclast length; OS/BS: osteoid surface by bone surface; RUNX2: Runt-related transcription factor 2; OSX: osterix; OCN: osteocalcin. Boxes and bars were median ± interquartile range. Mann-Whitney test: **p < 0.01 and ***p < 0.001.

Along with early bone loss, number of osteoclasts by bone perimeter (N.Oc/B.Pm), osteoclast surface by bone surface (Oc.S/BS) were enhanced from day 8, and osteoclast length (Oc.Le) from day10 in the AIA group compared to CTRL group (Fig. 3A–C, p < 0.01). These results were illustrated with representative histological slides of the navicular bone in CTRL and AIA (Fig. 3D,E). A similar pattern was observed in all ankle areas (data not shown). At the same time, the nonmineralized matrix deposit, as assessed by OS/BS, was strongly decreased in the rat AIA group versus CTRL group (Fig. 3F, p < 0.01) with representative slides (Fig. 3G,H) showing inhibition of osteoblastic synthesis. Expression of osteoblastic markers was significantly decreased in AIA compared to control from day 8 for Runt-related transcription factor 2 (RUNX2, p < 0.05) and from day 10 for osterix (OSX) and osteocalcin (OCN; Fig. 3I, p < 0.01 and p < 0.001, respectively).

### Gene expression for inflammation, osteoclastogenesis, and osteoclast activity were increased before AIA onset

**Figure 4:**
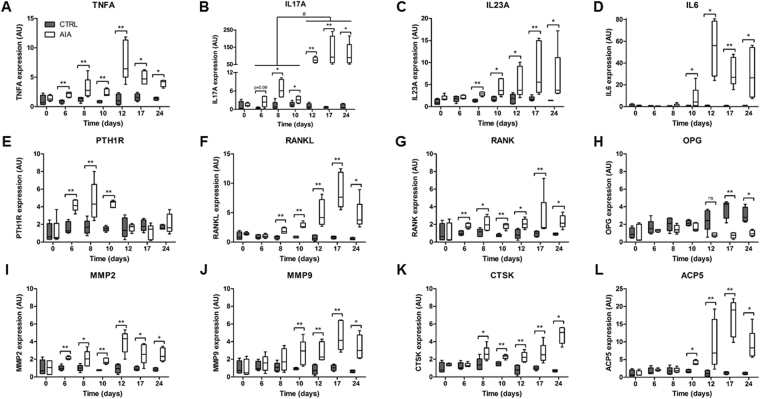
Early modifications in inflammation and resorption gene expression in the ankle. Quantitative RT-PCR was conducted at all 7 time points for evaluation of proinflammatory gene expression: (**A**) TNFA, (**B**) IL-17A, (**C**) IL-23A, and (**D**) IL-6. Expression of genes related to osteoclast activation pathway and matrix degradation function included (**E**) PTH1R, (**F**) RANKL, (**G**) RANK, (**H**) OPG, (**I**) MMP-2, (**J**) MMP-9, (**K**) CTSK, and (**L**) ACP5. Gene expression was normalized with housekeeping gene hypoxanthine-guanine phosphoribosyltransferase, and CTRL group expression at baseline was set at 1. Boxes and bars were median ± interquartile range. AU: arbitrary units; PTH1R: parathormone receptor 1; RANK: receptor activator of nuclear factor kappa B; RANKL: RANK ligand; OPG: osteoprotegerin; MMP: matrix metalloproteinase; CTSK: cathepsin K; ACP5: acid phosphatase 5, encoding the tartrate resistant acid phosphatase (TRAcP5). Kruskal-Wallis test in rat AIA group: ^#^p < 0.05. Mann-Whitney test: *p < 0.05, **p < 0.01, and ***p < 0.001.

In the ankle joint, overexpression of several proinflammatory cytokine genes started before arthritis onset, including TNFA from day 6 (Fig. 4A, p < 0.01) and IL-17A or IL-23A from day 8 (Fig. 4B,C, p < 0.05 and p < 0.01). Moreover, IL-17A was overexpressed (5-fold) before arthritis with a huge expression (55-fold) during the arthritis phase. IL-6 was strongly overexpressed from day 10 with a peak of expression at day 12 (60-fold) and remained high during arthritis (25-fold) (Fig. 4D, p < 0.05). Regarding osteoclast activation, osteoblast receptor PTH1R gene was increased only before arthritis onset in rat AIA (Fig. 4E, p < 0.01). RANKL expression was upregulated from day 8, while its osteoclast receptor RANK was increased earlier at day 6 and its decoy receptor OPG was decreased only at day 17 and day 24 (Fig. 4F–H, respectively, p < 0.01). After showing overexpression of osteoclast activation genes in rat AIA joint, we focused on matrix degradation markers. In the AIA joint, gelatinases MMP-2 and MMP-9 were upregulated from day 6 and day 10, respectively (Fig. 4I,J, respectively, p < 0.01), and CTSK and ACP5 from day 8 and day 10, respectively (Fig. 4K,L, p < 0.05).

### Inhibitors of bone formation were upregulated with different kinetics

**Figure 5:**
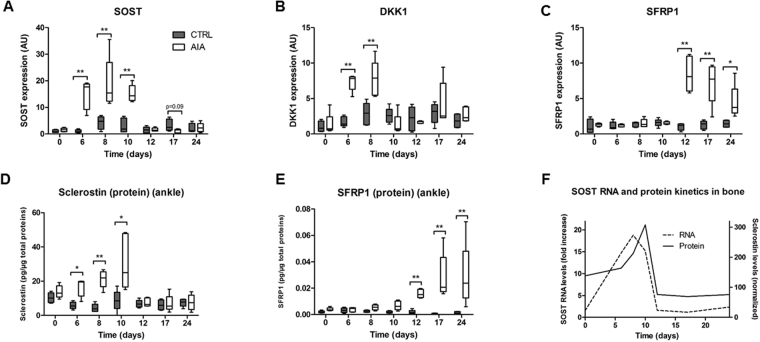
Early modifications in Wnt inhibitor expression. Quantitative RT-PCR for all 7 time points in ankle: (**A**) SOST, (**B**) DKK1, and (**C**) SFRP1. Protein assay in ankle at all 7 time points for (**D**) sclerostin and (**E**) SFRP1. Wnt inhibitors levels were normalized by total protein levels detected at the ankle site. (**F**) Timely comparison between SOST RNA and sclerostin protein levels in the ankle joint. Gene expression was normalized with housekeeping gene hypoxanthine-guanine phosphoribosyltransferase and CTRL group expression at baseline was set at 1. Boxes and bars were median ± interquartile range. AU: arbitrary units; DKK1: dickkopf-1; SFRP1: secreted frizzled related protein 1. Mann-Whitney test: *p < 0.05 and **p < 0.01.

In the AIA group compared to CTRL group, expression of Wnt inhibitors SOST and DKK1 was higher at the ankle site before arthritis only (Fig. 5A,B, p < 0.01 each), with a trend for a SOST downregulation at day 17 (p = 0.09). Contrarily, SFRP1 was higher in AIA compared to CTRL only after arthritis onset (Fig. 5C, p < 0.01). Furthermore, SOST and DKK1 gene expressions correlated together (Figure S3A, r = 0.49, p < 0.001), but not with SFRP1 gene expression (not shown). At the ankle level, higher sclerostin concentration was observed at the protein level in the AIA group compared to CTRL group from day 6 to 10 (Fig. 5D, p < 0.05), while SFRP1 protein expression was only enhanced after arthritis onset (Fig. 5E, p < 0.01). Ankle sclerostin protein concentration correlated with SOST RNA expression. Moreover, RNA SOST peak preceded protein sclerostin peak in the ankle from rat AIA (for SOST/sclerostin) (Fig. 5F, p < 0.05 and Figure S3B, r = 0.36, p < 0.01). Focusing on day 8, circulating sclerostin concentration was higher in rat AIA compared to CTRL (Figure S3C, p < 0.01) and correlated with its ankle concentration (Figure S3D, r = 0.70, p < 0.05). Non-canonical WNT5A expression was also modestly altered at day 8 (Figure S3E, p < 0.05).

### Inhibition of bone formation correlated with SOST in early arthritis and with SFRP1 in late arthritis

**Figure 6:**
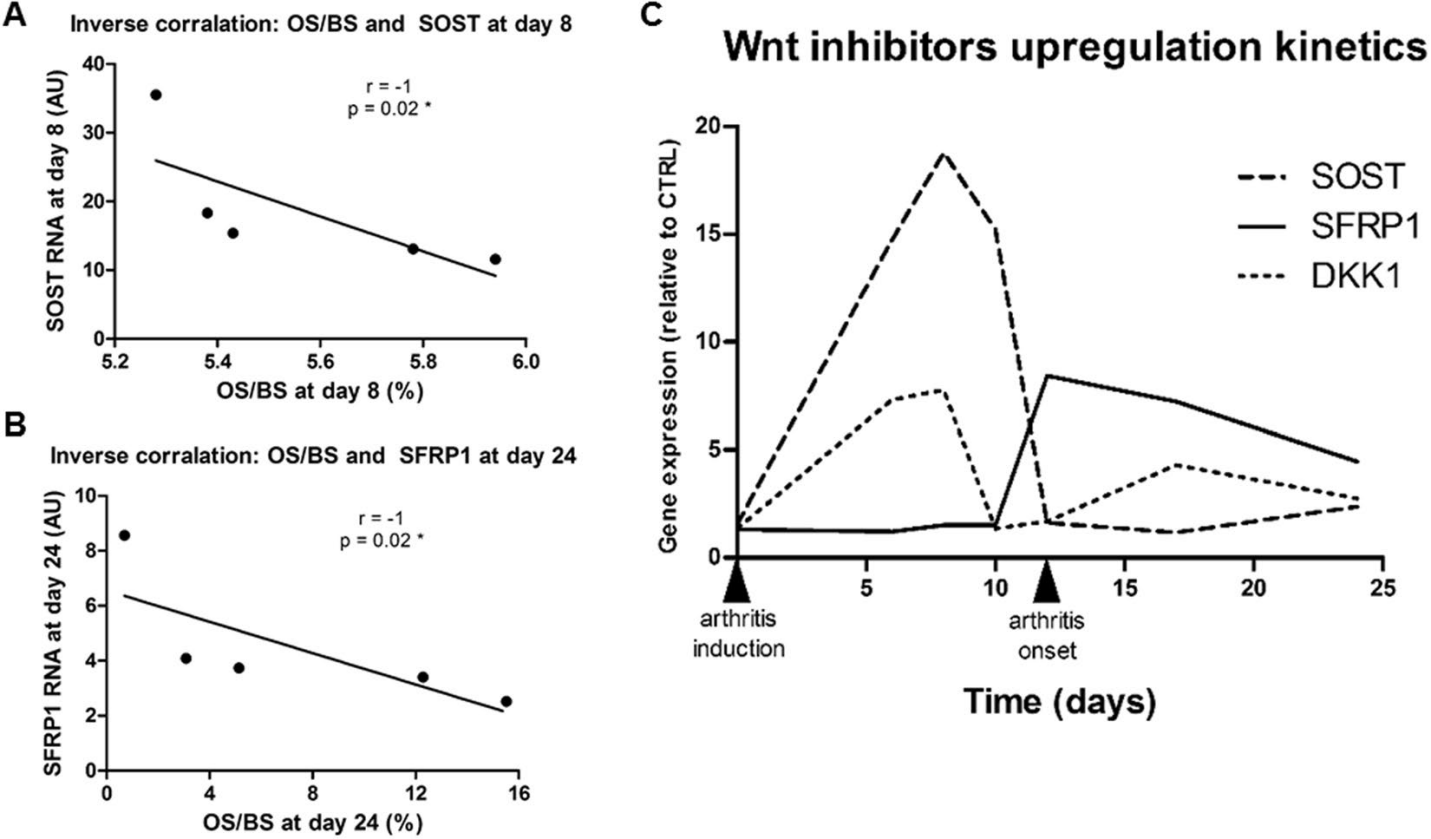
Loss of OS/BS correlated with SOST at day 8, but with SFRP1 at day 24. Spearman correlations were performed between OS/BS and (**A**) SOST at day 8 and (**B**) SFRP1 at day 24. (**C**) Relative expression of SOST, SFRP1, and DKK1 over time in AIA group compared to CTRL group. Bimodal kinetics with SOST and DKK1 highly expressed before arthritis onset followed by high SFRP1 expression only after arthritis onset. Furthermore, SOST peak relative expression to CTRL was dominant compared to DKK1 or SFRP1. OS/BS: osteoid surface/bone surface; AU: arbitrary units; DKK1: dickkopf 1; SFRP1: secreted frizzled related protein 1. Spearman correlations; r: Spearman coefficient, *p < 0.05.

Correlations between histological markers and gene expressions were performed in the AIA group. Before arthritis onset at day 8, OS/BS was inversely correlated with SOST (Fig. 6A, r = −1, p < 0.02) but not with DKK1 or SFRP1 (Table S2, r = −0.50, p = 0.45 for both), suggesting that high SOST at day 8 correlated with low osteoblast activity. However, at day 24, OS/BS was inversely correlated with SFRP1 (Fig. 6B, r = −1, p < 0.02) but not with SOST or DKK1 (Table S2, SOST: r = −0.50, p = 0.45, and DKK1: r = −0.70, p = 0.23). As expected, resorption parameter N.Oc/B.Pm correlated with CTSK and ACP5 at day 8 (Table S2, r = 1, p = 0.02 for both). Thus, high CTSK and ACP5 in AIA rats were correlated with high osteoclast activity. Taken together, these results suggested that inhibition of bone formation was more likely to be explained by SOST in early arthritis and by SFRP1 in late arthritis, suggesting a switch in the role of Wnt inhibitors (Fig. 6C). We confirmed that SOST and DKK1 at day 8 strongly correlated with TNFA and IL23A overexpression (Table S3, p < 0.001 all). SFRP1 expression at day 17 correlated with elevated IL6 and IL17A (p < 0.01 both). The late decrease of SOST was not related to osteocyte apoptosis induced by arthritis, as according to H&E histology, osteocyte density and lacunae occupancy remained the same at day 17 (Figure S3F and G).

## Discussion

Previous insights suggested that bone loss could begin earlier than expected during arthritis^7,9,10^. Here we showed that cortical and trabecular microstructure deteriorated before arthritis onset. The bone microarchitecture alteration was related to a strong decrease in bone osteoblastic activity and increased bone destruction, mainly after arthritis onset. The early bone degradation before arthritis onset reinforced the Inside-Outside hypothesis^7,8^.

In humans, exploration of preclinical RA is difficult in particular for mechanistic approaches. RA onset is defined by joint effusion onset^2^. Before this onset, some abnormalities were reported including ACPA development, but the first joint effusion was not predictable. So, a robust animal model with high arthritis rate and synchronicity was required to explore the preclinical phase. As we showed previously, the rat AIA model met these requirements^12^. A previous study using the same model reported increased osteoclast activity at the time of arthritis and later^26^. With µCT, we observed the first abnormalities before arthritis onset at day 8 involving cortical and trabecular compartments. Cortical porosity was increased with a decrease in bone surface fraction with a reduction in number and thickness of trabeculae. Then bone histomorphometry analysis provided evidence that osteoclastic surfaces already increased before arthritis onset associated with a reduction in osteoblastic activity. Quantification of osteoblast parameters was performed indirectly by using quantification of osteoblastic transcript due to low reproducibility of osteoblast count in small bones of ankle. We then explored mechanisms explaining this imbalance before arthritis onset. Proinflammatory cytokines at the ankle level were involved as previously described from the arthritis onset^27^. We confirmed previous data with early involvement of TNF followed by IL-17A and IL-23 before arthritis onset followed by IL-6^27^. After arthritis onset, IL-17A was strongly upregulated, suggesting an adaptative response after its innate response in prearthritis phase. All these cytokines are strong inducers of osteoclastogenesis through the RANK-RANKL pathway^28^. Thus, early TNF upregulation at day 6 could explain high TRAcP5 positive cells at day 8. We also provided data for another pathway able to induce RANKL. In fact, PTH1R after binding to its ligand PTH can induce RANKL as confirmed by our kinetic studies^29–31^. We also observed an early activation of CTSK from day 8 along with early bone damage. Despite an early TRAcP5 staining from day 8, its ACP5 RNA increased with a delay of 2 days. This might be explained by a pro-TRAcP5 inactive form that is stored in osteoclasts after translation and cleaved with a strong TRAcP5 activity and its RNA later increased to renew the stock^32,33^. Furthermore, active suppression of the counterbalance OPG was not involved in early arthritis. Here, we reported upregulation of MMP-2 and MMP-9 genes from day 6 and day 10, respectively, as previously reported^34^. MMP-9 kinetic expression was similar to IL-6 kinetic expression, which was reported as its strong activator^35^.

After confirming huge osteoclast activation, we focused on gene expressions of bone formation inhibitors before and after arthritis onset at the ankle site. We demonstrated that DKK1 and sclerostin were only upregulated before arthritis onset, while SFRP1 was only upregulated after arthritis onset. Furthermore, we showed that systemic SOST concentration correlated with local ankle expression for the first time at day 8. So, sclerostin serum level could be proposed as a biomarker of arthritis onset^15–18^. These results provide serious insights for the paradigm of sclerostin inhibition during arthritis and suggest that inhibitor kinetics have to be evaluated in all animal models. Anyway, an early inhibition of sclerostin appeared to be effective on bone loss at the local and systemic sites^21^. In the French ESPOIR cohort of early RA, serum DKK1 correlated with inflammation parameters and DAS28, whereas serum sclerostin concentration correlated with RA demographic parameters^18^. Sclerostin blood concentration was not higher in established RA patients than in control subjects but still correlated with low bone mineral density^36^. Here, we observed that SOST, but not DKK1 correlated with the early loss of OS/BS. Accordingly, cells producing SOST, such as osteocytes, were responsible for mineralization decrease, while cells producing DKK1 should have other roles. Sclerostin is mainly produced by mature osteocytes^37^ and hypertrophic chondrocytes^38^, whereas DKK1 is ubiquitously produced by many cell types including synoviocytes^39^. We also observed a strong correlation between SOST expression and sclerostin concentration at local and systemic levels. In our study, we observed correlations between SOST or DKK1 and TNFA or IL23A, in accordance with previous studies by others in the bone or in the synovium^40,41^. The third Wnt inhibitor investigated, SFRP1, was highly expressed only after arthritis onset in the AIA model. We identified the correlation of SFRP1 with IL17A and IL6. SFRP1 was once associated with the Th17 response^25^, but its links in synovium and bone with IL6 remain to be investigated. Targeting this Wnt inhibitor could be interesting in arthritis treatment and easier than sclerostin or DKK1 because its window of upregulation is in declared arthritis. Osteocytes exhibit a leading role in bone regulation^42^. They are potent inducers of RANKL and sclerostin for bone resorption^43,44^ and formation inhibition^37^, respectively. The low osteoblast activity correlated with sclerostin in early arthritis and SFRP1 in established arthritis. Compared to sclerostin, SFRP1 inhibits directly Wnt and Frizzled^45^ instead of coreceptor LRP5/6, and thus might be more powerful. Our data with up regulation of SFRP1 after arthritis onset are not consistent with previous data from human samples. In human, SFRp1 was observed down regulated in fibroblast like synoviocytes from RA compared to them from osteoarthritis^46,47^. To these classic Wnt regulators, the role of non-canonical mediators has to be clarified, such as WNT5A, described as a bone turnover enhancer^48,49^.

The main limitation of our data was the absence of replication in another model. However, animal models with 100% of arthritis onset at the same time are limited. Although the total animal number in our study was large, only 5 animals were analysed at each time point. Despite this small number at each time point, we were able to detect difference on histology and µCT due to the very reproducible model.

Taken altogether, our results reinforced the demonstration of an active role of the bone compartment in AIA. The monitoring of bone marrow cell populations, especially the immune cells and their ability to regulate the Wnt pathway remains to be investigated. Correlation between Wnt inhibitors and inflammatory markers along arthritis suggested an immune-mediated regulation; further mechanisms need to be investigated in the future, especially in human tissues.

## Methods

### Animals and ethics

This study was performed in accordance to the legislation of the European Community and was approved by the Ethical Committee for Animal Experiments of Saint-Etienne University (registered to the French government as #0125.01). All methods were performed in accordance with these guidelines and regulations. Six-week-old female Lewis rats (Janvier Laboratories, Saint-Germain-sur-l’Arbresle, France) were housed in the PLEXAN facility with 12/12 h light/dark cycles and *ad libitum* water and food access. After 7 days of housing and acclimation training, rats were randomly separated into two groups. In the rat AIA (n = 35), arthritis was induced by subcutaneous injection of 300 µL of 5 mg.mL^−1^ *Mycobacterium butyricum* (Difco Laboratories, Detroit, MI, US) in mineral oil. Control rats (CTRL group, n = 35) received only mineral oil. Injection was performed under short and light 2% isoflurane anaesthesia to optimize the full deliverance of the dose and obtain a high rate of arthritis induction^12^. The day of arthritis induction was defined as day 0. Seven time points were defined: 0, 6, 8, 10, 12, 17, and 24 days. Five AIA and 5 CTRL rats were sacrificed at each time point to perform histological and biochemical analysis.

### Arthritis monitoring

*In vivo* follow-up included animal care and appreciation of overall welfare. Body mass, AI, and ankle circumferences were recorded as previously described^12^. Briefly, AI was measured by a blinded observer and summarized clinical inflammation status, mainly erythema; while ankle circumference delivered a quantitative assessment of joint swelling. However, we previously observed that in chronic arthritis, remission of erythema and oedema were not correlated to gain of limb function or improvement of the gait. In other words, AI and ankle circumference could not totally sum up the arthritis grade of the subject. Consequently, we developed a new complementary scoring of lameness of hindlimb, named LFI. LFI is calculated from 0 to 4 as follows: 0: regular gait; 1: intermittent lameness (intermittent stop of use of the proximal half of the paw: first discomfort in the ankle); 2: continuous lameness (only the metatarsals are used: arthritis in the ankle area); 3: continuous lameness with altered positioning of the paw (preferential use of the top of the paw by bending the metatarsals: arthritis reaches proximal metatarsals); 4: complete loss of function (the paw is not used anymore: severe arthritis in all the paw).

### Microcomputed tomography (µCT)

Right ankles were scanned *ex vivo* with the µCT vivaCT40 (Scanco, Brütisellen, Switzerland) at 55 kVp (peak kilovoltage) and reconstructed under a resolution of 12.5 µm. Porosity and erosions were qualitatively detected on 2-D slices, while quantification and 3-D imaging were performed after reconstruction. Reconstruction was performed under 1.2/2/260 for the cortical layer and under 2.8/2/289 for the trabecular network (Gauss sigma/Gauss support/lower threshold).

### Undecalcified bone histomorphometry and histoenzymology

Right ankles were fixated in 10% paraformaldehyde for 48 h. After µ-CT assessment in ethanol, they were progressively dehydrated in 50% acetone for 48 h and 100% acetone for 1 week. Embedding in methylmethacrylate plastic was performed by successive impregnations at −20 °C, and polymerisation at 5 °C. Parasagittal slices (9 µm) were obtained with SM2500E microtome (Leica, Wetzlar, Germany). Inflammatory infiltrate was assessed with hematoxylin and eosin staining. Briefly, slices were stained by filtered Mayer hematoxylin for 10 min, washed, exposed to eosin for 5 min, washed, and assembled. As previously described^12^, TRAcP5 staining was performed for assessment of osteoclast activity and microarchitecture 2-D calculation allowing the determination of the osteoclast number by bone perimeter (N.Oc/B.Pm), the ratio of osteoclastic surface to bone surface (Oc.S/BS), and osteoclast length (Oc.Le). These bone cellular and macroscopic parameters were measured with a semiautomatic system: digitizing tablet (Summasketch; Summagraphics, Paris, France) connected to a PC with software designed in our Laboratory (Histomorphometrie osseuse animale 2.11). Modified Goldner trichrome staining was performed for mineralization observation and calculation of the osteoid surface by bone surface (OS/BS) parameter. Slices were observed with a Leica DMRB microscope using the Bone Morpho software (Exploranova, La Rochelle, France).

### RNA analysis

Left ankles were dissected under sterile conditions, frozen in liquid nitrogen, and then stored at −80 °C until extraction. Tissue lysis was performed in TRI Reagent (Sigma, Saint Louis, MO, US). RNA was separated from proteins after centrifugation in chloroform. RNA was purified with RNeasy plus (Qiagen, Venlo, Netherlands). Quality and quantity of RNA were assessed by Experion RNA analysis (BioRad, Hercules, CA, US) and QuantIT RiboGreen RNA assay (Thermo Scientific, Waltham, MA, US), respectively. Reverse transcription (RT) was performed on 2 µg of RNA. Quantitative RT polymerase chain reaction (PCR) was conducted on CFX96 RealTime System (BioRad) with LightCycler FastStart DNA Master plus SYBRgreen I (Roche Diagnostics, Basel, Switzerland). Primer sequences of candidate genes are detailed in Supplementary Table S1. The results were normalized to the housekeeping gene expression hypoxanthine-guanine phosphoribosyltransferase and presented as the variation in fold of gene expression compared to CTRL group at day 0.

### Protein analysis

After chloroform separation, the protein fraction was purified with isopropanol, chlorhydrate guanidine, and ethanol. After ultrasound exposure, samples were homogenised in 1% SDS and stored with protease inhibitors. Quantification of total proteins was performed with Pierce BCA protein assay (Thermo Scientific). Sclerostin (R&D systems, Minneapolis, MN, US) and SFRP1 protein (Cusabio, College Park, MD, US) were quantified at the ankle site and in the serum by using antibody-based detection assays (R&D systems, Minneapolis, MN, US (R&D systems, Minneapolis, MN, US and Cusabio, College Park, MD, US, respectively).

### Statistical analysis

Nonparametric statistical analysis was performed. The results were presented as median and interquartile range IQR. Mann-Whitney test was applied to compare CTRL group and AIA group at one time point. Kruskal-Wallis test was used for comparison from day 0 to day 24, and significant differences between two time points were assessed with Dunns post-test. Correlation was assessed by Spearman correlation test. P-values less than 0.05 were considered statistically significant.

## Electronic supplementary material

Supplementary data set
