# Supplementary material for: Early sclerostin expression explains bone formation inhibition before arthritis onset in the rat adjuvant-induced arthritis model
Source: Sci Rep. 2018 Feb 22;8:3492. doi: 10.1038/s41598-018-21886-w (PMC5823923; doi:10.1038/s41598-018-21886-w)
Supplement: Supplementary file 1 — Supplementary data set [file 41598_2018_21886_MOESM1_ESM.doc]

**Early sclerostin expression explains bone formation inhibition before arthritis onset in the rat adjuvant-induced arthritis model**

Guillaume Courbon^1^, Raphaëlle Lamarque^1^, Maude Gerbaix^1^, Robin Caire^1^, Marie-Thérèse Linossier^1^, Norbert Laroche^1^, Mireille Thomas^1^, Thierry Thomas^1,2^, Laurence Vico^1^, Hubert Marotte^1,2,*^

^1^SAINBIOSE, INSERM U1059, University of Lyon, Saint-Etienne, France

^2^Department of Rheumatology, Hopital Nord, University Hospital, Saint-Etienne, France

**Supplemental Figure 1**

**
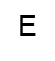

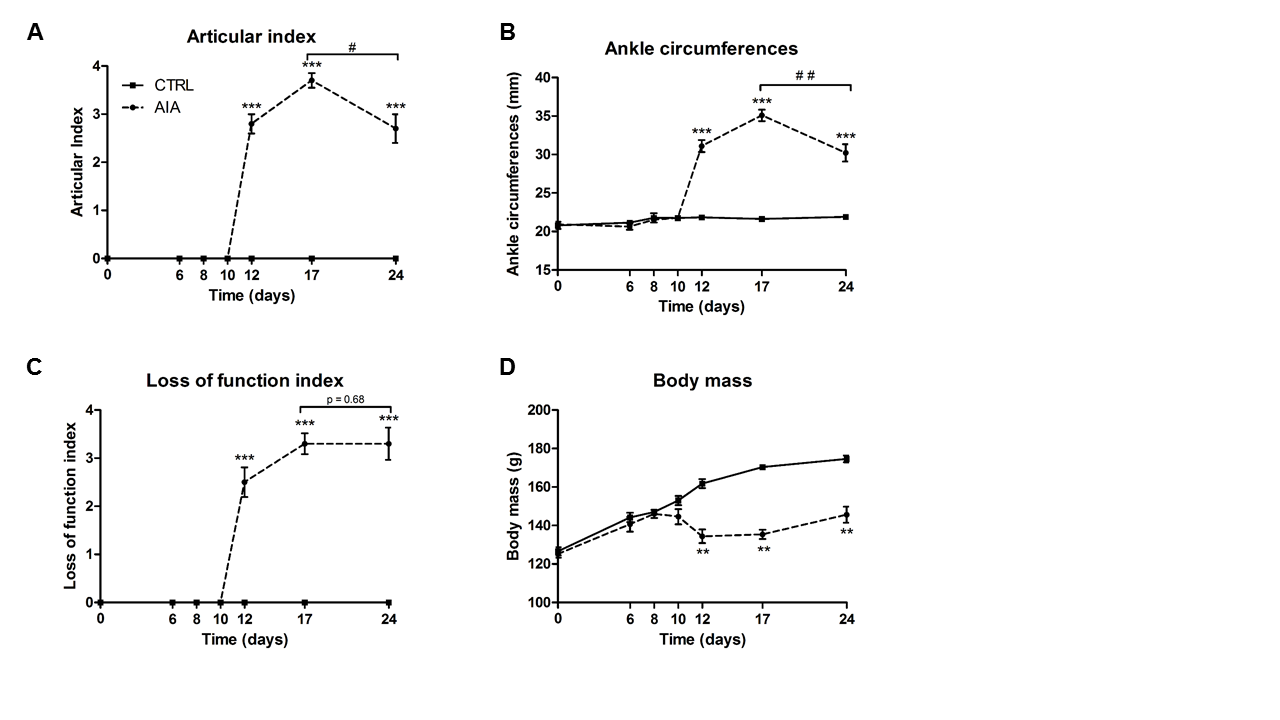
**

**
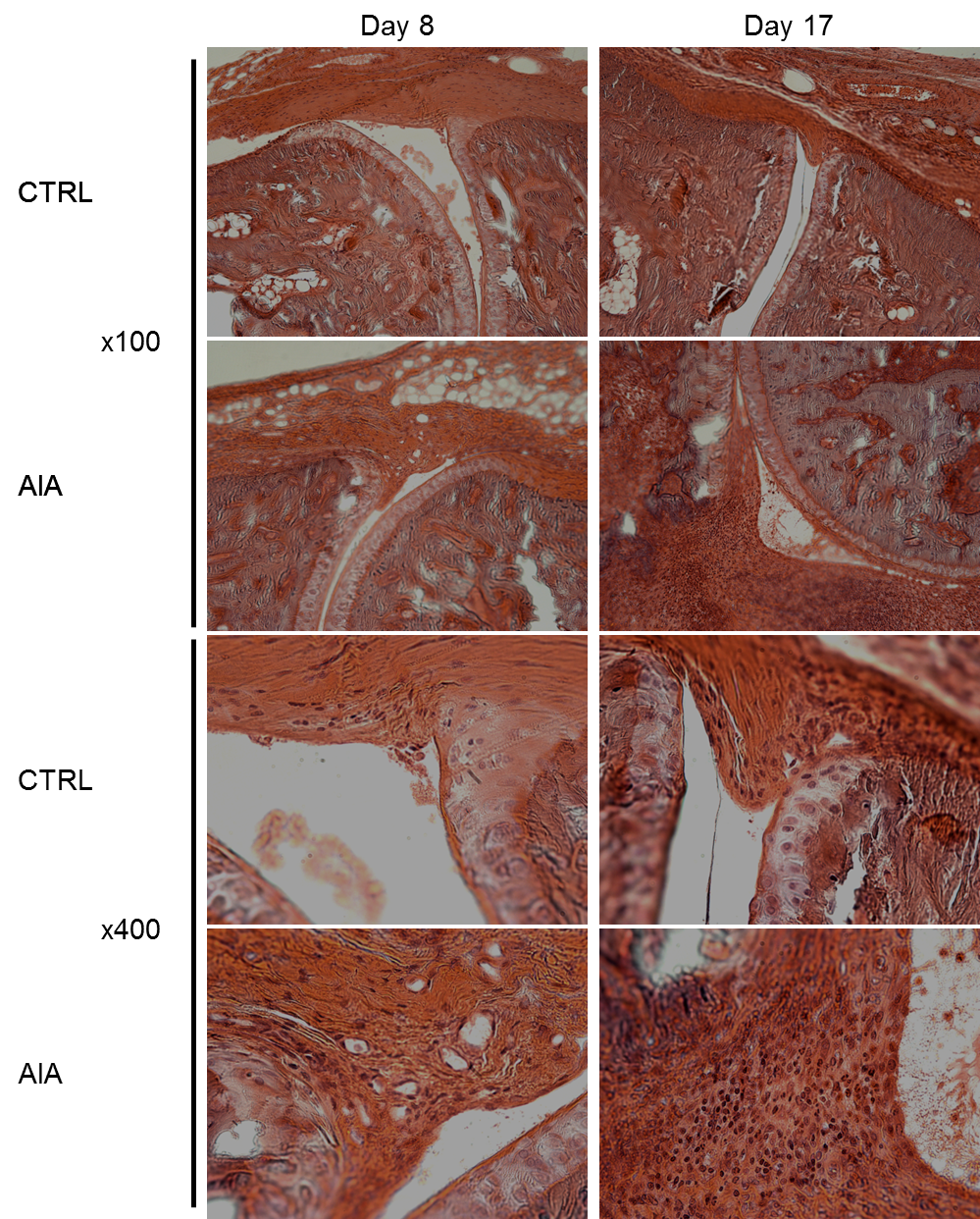
**

**Supplemental Figure 2**

**
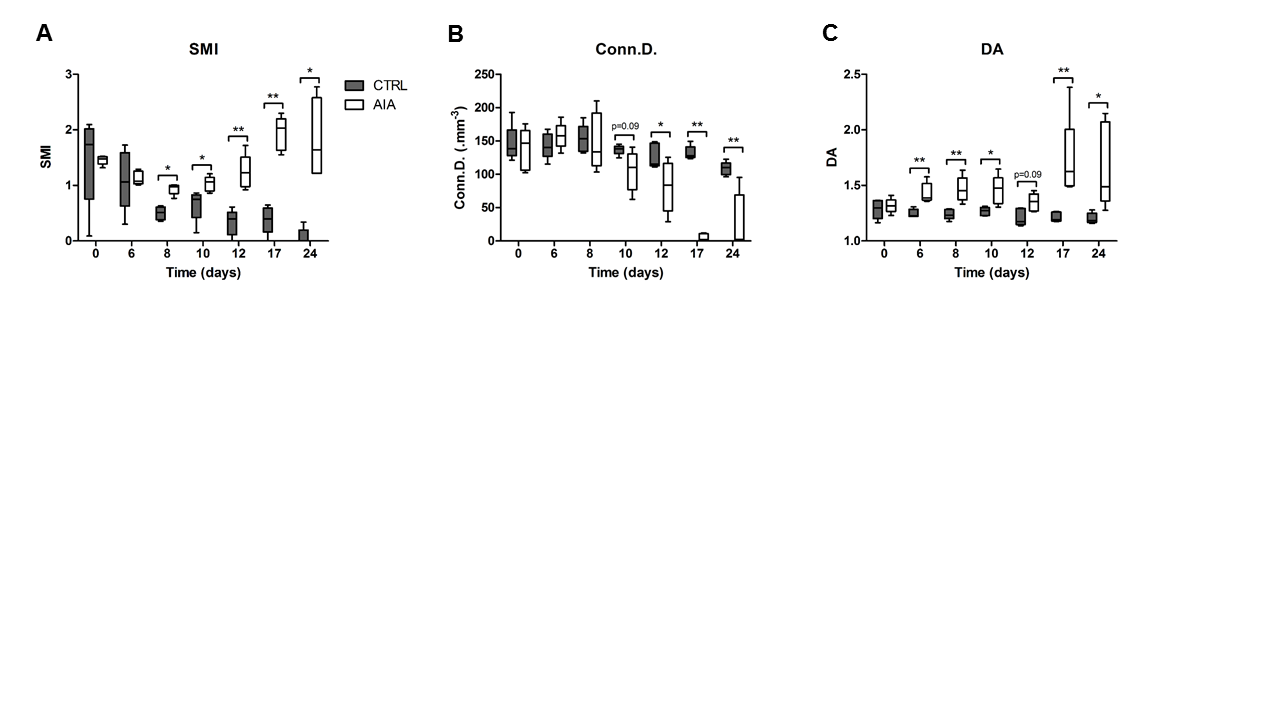
**

**Supplemental Figure 3**

**
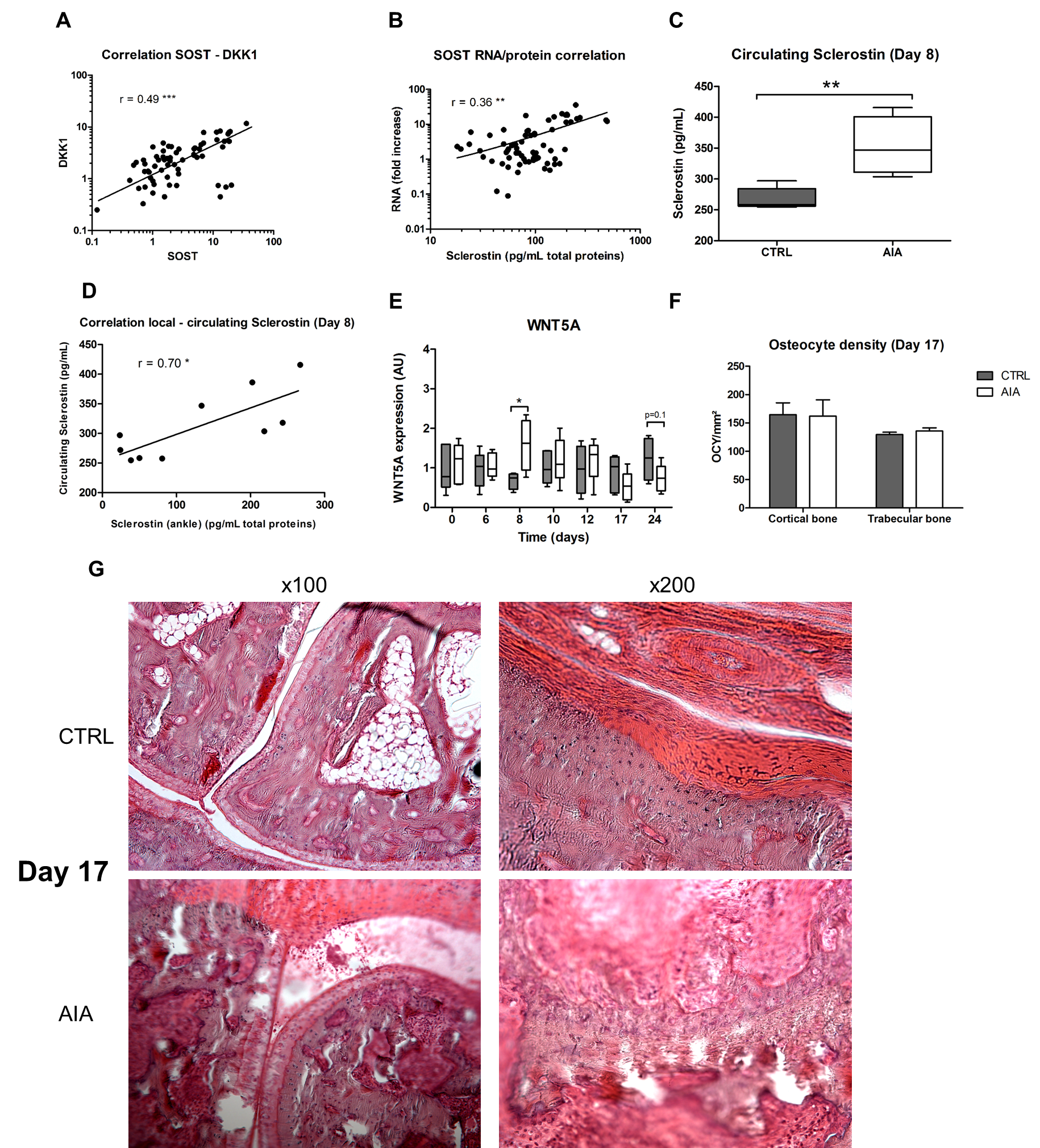
**

**Figure Legends**

**Figure S1.** Joint inflammation was detected at day 12 during these experiments. First clinical signs at day 12 included: (A) articular index, (B) ankle circumferences, (C) loss of function index, and (D) body mass. (E) Hematoxylin and eosin cell staining on undecalcified histological slices (talo-navicular joint) at day 8 (left). Day 17 was used as a positive control (right). Scale bar: 500µm. AIA: adjuvant-induced arthritis (dash line/circles); CTRL: control (solid line, squares). Circles and squares were medians and bars were SEM. Kruskal-Wallis test: ^#^p<0.05 and ^##^p<0.01. Mann-Whitney test: **p<0.01 and ***p<0.001.

**Figure S2.** Extended analysis of the trabecular structure of the navicular bone confirmed early bone alteration in AIA. (A) SMI: structure model index from 0 (dense network of elements with several long dimensions) to 3 (loose network of elements with small dimensions). (B) Conn.D.: connectivity density, in number of connections by cm^3^. (C) DA: degree of anisotropy change, distorted in AIA with different orientation compared to structure orientation in CTRL. Boxes and bars were median and interquartile range. AIA: adjuvant-induced arthritis; CTRL: control. Mann-Whitney test: *p<0.05 and **p<0.01.

**Figure S3.** Correlation assessment of Wnt inhibitors and early detection of circulating sclerostin. Correlations (A) between SOST RNA and DKK1 RNA over all experiment, (B) between SOST RNA and sclerostin protein over all experiments. (C) Circulating sclerostin protein levels in the serum at day 8. (D) Correlation between local and circulating sclerostin levels at day 8. (E) Non-canonical Wnt activator WNT5A mRNA expression. (F) Osteocyte density at day 17 in the navicular bone, stained with H&E. No significant differences were observed in cortical and trabecular densities. (G) Hematoxylin and eosin staining of osteocyte and lacunae at day 17. No difference in empty lacunae was observed. Spearman correlations; r: Spearman coefficient, except for (C, E): Mann-Whitney test. *p<0.05, **p<0.01, and ***p<0.001. DKK1: dickkopf 1. OCY: osteocyte.

**Table S1**: **PCR primer sequences in 5’-3’ direction**

| Target | Species | Primers | Tm (°C) | Product size (bp) | Source |
| --- | --- | --- | --- | --- | --- |
| TNFA | Rat | Forward: cacgctcttctgtctactga  Reverse: gtaccaccagttggttgtct | 63.0 | 254 | NM_ 012675.3 |
| IL17A | Rat | Forward: gaaggcagcggtactcatcc  Reverse: cctcattgcggctcagagtc | 63.0 | 182 | NM_00110697.1 |
| IL23A | Rat | Forward: cacctgctggactcggacat  Reverse: ttggaggctgcgaaggatct | 63.0 | 235 | NM_130410.2 |
| IL6 | Rat | Forward: ttccagccagttgccttctt  Reverse: cagtgcatcatcgctgttca | 63.0 | 225 | NM_012589.1 |
| PTH1R | Rat | Forward: cagcgagtgcctcaagttca  Reverse: gatgtagttgcgcgtgcagt | 63.0 | 166 | NM_020073.2 |
| RANKL | Rat | Forward: gacagcacgcgctgcttcta  Reverse: ccacatcgagccacgaacct | 63.0 | 220 | NM_057149.1 |
| RANK | Rat | Forward: atcgtcctgctcctcttcat  Reverse: acttcttgctggctggagtt | 64.0 | 200 | NM_001271235.1 |
| OPG | Rat | Forward: gagtgtgcgaatgtgaggaa  Reverse: aattagcaggaggccaagtg | 63.0 | 215 | NM_012870.2 |
| MMP-2 | Rat | Forward: gagttggcagtgcaatacct  Reverse: catggtctcgatggtgttct | 63.0 | 150 | NM_031054.2 |
| MMP-9 | Rat | Forward: ttcgacgacgacgagttgtg  Reverse: tgccatgctccgtgtagaga | 63.0 | 230 | NM_031055.1 |
| CTSK | Rat | Forward: agtgccaccttcgcgttcct  Reverse: tagccgcctccacagccata | 63.0 | 261 | NM_031560.2 |
| ACP5 | Rat | Forward: cagccaaggaggactatgtt  Reverse: acaccgttctcatcctgaag | 63.0 | 178 | NM_019144.2 var 1 |
| SOST | Rat | Forward: gcaccatgcagctctcacta  Reverse: gctgtactcggacacgtctt | 63.0 | 242 | NM_030584.1 |
| DKK1 | Rat | Forward: ctgcatgaggcacgctatgt  Reverse: ctgtggcgcagtctgatgat | 63.0 | 236 | NM_001106350.1 |
| SFRP1 | Rat | Forward: acgtctgcatcgccatgacc  Reverse: acgagccgcttcagctcctt | 63.0 | 247 | NM_001276712.1 |
| HPRT1 | Rat | Forward: gttggatacaggccagactt  Reverse: gccacatcaacaggactctt | 63.0 | 188 | NM_012583.2 |
| RUNX2 | Rat | Forward: cagaccagcagcactccata  Reverse: cgccagacagactcatccat | 63.0 | 245 | NM_001278483.1 var 1 |
| OSX | Rat | Forward: gtacggcaaggcttcgcatc  Reverse: cgcttggagcagagcagaca | 63.0 | 175 | NM_001037632.1 var 1 |
| OCN | Rat | Forward: caactcggtgcagacctagc  Reverse: gaggtagcgccggagtctat | 63.0 | 180 | NM_013414.1 |
| WNT5A | Rat | Forward: cttgggcacatttccacgcta  Reverse: attacaacctgggcgaaggag | 63.0 | 340 | NM_022631.2 |

**Table S2.** Correlation between formation inhibition observed in histology and gene expression in AIA rats.

|  | Histological parameter | RNA marker | r _(Spearman)_ | p-value |
| --- | --- | --- | --- | --- |
| Formation  inhibition  at day 8 | OS/BS | **SOST** | **-1** | **0.02 *** |
|  |  | DKK1 | -0.50 | 0.45 |
|  |  | SFRP1 | -0.50 | 0.45 |
| Formation  inhibition  at day 24 | OS/BS | SOST | -0.50 | 0.45 |
|  |  | DKK1 | -0.70 | 0.23 |
|  |  | **SFRP1** | **-1** | **0.02 *** |
| Resorption  activation  at day 8 | N.Oc/B.Pm | RANKL | 0.70 | 0.23 |
|  |  | RANK | 0.90 | 0.08 |
|  |  | MMP2 | 0.90 | 0.08 |
|  |  | MMP9 | -0.10 | 0.95 |
|  |  | **CTSK** | **1** | **0.02 *** |
|  |  | **ACP5** | **1** | **0.02 *** |

Formation parameter OS/BS was inversely correlated with SOST at day 8 and SFRP1 at day 24. Resorption parameter N.Oc/B.Pm was correlated with CTSK and ACP5. AIA: adjuvant-induced arthritis. OS/BS: osteoid surface/bone surface, N.Oc/B.Pm: number of osteoclasts/bone perimeter, DKK1: dickkopf 1, SFRP1: secreted frizzled related protein 1, RANKL: RANK ligand, RANK: receptor activator of NFκB, MMP: metalloproteinase, CTSK: cathepsin K, ACP5: acid phosphatase 5. Spearman correlations with r coefficient and p-value. *p<0.05; ***p<0.001.

**Table S3.** Correlations between inflammatory markers and Wnt inhibitors along arthritis.

|  | Histological parameter | RNA marker | r _(Spearman)_ | p-value |
| --- | --- | --- | --- | --- |
| Day 8 | SOST | TNFA | 0.88 | <0.001 *** |
|  |  | IL23A | 0.90 | <0.001 *** |
|  | DKK1 | TNFA | 0.96 | <0.001 *** |
|  |  | IL23A | 0.93 | <0.001 *** |
| Day 17 | SFRP1 | IL6 | 0.77 | 0.009 ** |
|  |  | IL17A | 0.78 | 0.008 ** |

Spearman correlations for SOST and DKK1 with TNFA and IL23A at day 8, and for SFRP1 and IL6 and IL17A at day 17. DKK1: dickkopf 1, SFRP1: secreted frizzled related protein 1. Spearman correlations with r coefficient and p-value. **p<0.01; ***p<0.001.
